# 3D amide proton transfer-weighted imaging may be useful for diagnosing early-stage breast cancer: a prospective monocentric study

**DOI:** 10.1186/s41747-024-00439-z

**Published:** 2024-04-08

**Authors:** Yeqin Li, Yan Zhang, Liwen Tian, Ju Li, Huihua Li, Ximing Wang, Cuiyan Wang

**Affiliations:** 1 Department of Radiology, Shandong Province Hospital of Traditional Chinese Medicine, Jinan, 250014 China; 2 Department of Radiology, Shandong Provincial Hospital Affiliated to Shandong First Medcial University, Jinan, 250021 China; 3 Department of Radiology, Shandong Public Health Clinical Center, Jinan, 250100 China; 4 Binzhou Medical University, Yantai, 264003 China

**Keywords:** Amides, Breast neoplasms, Early detection of cancer, Magnetic resonance imaging, Ki-67 antigen

## Abstract

**Background:**

We investigated the value of three-dimensional amide proton transfer-weighted imaging (3D-APTWI) in the diagnosis of early-stage breast cancer (BC) and its correlation with the immunohistochemical characteristics of malignant lesions.

**Methods:**

Seventy-eight women underwent APTWI and dynamic contrast-enhanced (DCE)-MRI. Pathological results were categorized as either benign (*n* = 43) or malignant (*n* = 37) lesions. The parameters of APTWI and DCE-MRI were compared between the benign and malignant groups. The diagnostic value of 3D-APTWI was evaluated using the area under the receiver operating characteristic curve (ROC-AUC) to establish a diagnostic threshold. Pearson’s correlation was used to analyze the correlation between the magnetization transfer asymmetry (MTR_asym_) and immunohistochemical characteristics.

**Results:**

The MTR_asym_ and time-to-peak of malignancies were significantly lower than those of benign lesions (all *p* < 0.010). The volume transfer constant, rate constant, and wash-in and wash-out rates of malignancies were all significantly greater than those of benign lesions (all *p* < 0.010). ROC-AUCs of 3D-APTWI, DCE-MRI, and 3D-APTWI+DCE to differential diagnosis between early-stage BC and benign lesions were 0.816, 0.745, and 0.858, respectively. Only the difference between AUC_APT+DCE_ and AUC_DCE_ was significant (*p* < 0.010). When a threshold of MTR_asym_ for malignancy for 2.42%, the sensitivity and specificity of 3D-APTWI for BC diagnosis were 86.5% and 67.6%, respectively; MTR_asym_ was modestly positively correlated with pathological grade (*r* = 0.476, *p* = 0.003) and Ki-67 (*r* = 0.419, *p* = 0.020).

**Conclusions:**

3D-APTWI may be used as a supplementary method for patients with contraindications of DCE-MRI. MTR_asym_ can imply the proliferation activities of early-stage BC.

**Relevance statement:**

3D-APTWI can be an alternative diagnostic method for patients with early-stage BC who are not suitable for contrast injection.

**Key points:**

• 3D-APTWI reflects the changes in the microenvironment of early-stage breast cancer.

• Combined 3D-APTWI is superior to DCE-MRI alone for early-stage breast cancer diagnosis.

• 3D-APTWI improves the diagnostic accuracy of early-stage breast cancer.

**Graphical Abstract:**

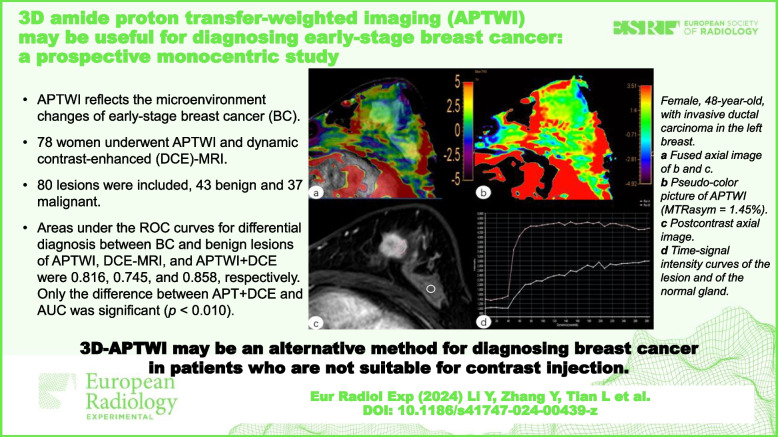

## Background

Breast cancer (BC) is the most common malignancy in women. According to the latest global cancer data [1], the global incidence of BC in women was 24.5% in 2020, with up to 2.26 million new cases. This exceeded the number of pulmonary carcinomas (2.20 million). Meanwhile, BC has the highest morbidity rate in China, making up 19.9% of new cancer cases.

Despite the high incidence of BC, its survival rate has significantly increased over the past few decades. Global cancer survival monitoring data show that the 5-year survival rate of breast cancer patients in China has increased to 83.2% [2]. However, the 5-year survival rate for patients with advanced-stage breast cancer is only 20% [3]. Therefore, early detection and diagnosis of BC are enormously important for treatment and prognosis.

Magnetic resonance imaging (MRI) plays an increasingly important role in the examination of BC. Due to its superior soft-tissue resolution, non-invasiveness, and absence of radiation exposure [4], contrast-enhanced breast MRI has gradually become a relevant imaging method for the diagnosis of breast diseases, with high sensitivity [5, 6]. However, all commonly used MR scanning sequences have limitations. For example, the plain scanning sequence can reflect macroscopic features of lesions, such as size and shape, but cannot effectively assess microscopic information about cells and blood supply. Additionally, the partial overlap of enhancement modalities and time-intensity curves between benign and malignant lesions, leads to not very satisfactory specificity in diagnosis of DCE-MRI, especially for early-stage breast cancer and benign lesions [7, 8]. In addition, linear-gadolinium-based MRI contrast agents are known to accumulate in the body [9], and the effects of this progression build-up are not completely known.

In response to these limitations, several new MRI technologies have emerged in recent years. Amide proton transfer-weighted imaging (APTWI) is a quantitative technique that can noninvasively detect amide protons in endogenous low-concentration proteins and peptides, reflecting their concentration and environmental changes [10]. There is a complex amide proton resonance at approximately 3.5 ppm down-field from the water resonance. In APTWI, the protons in free water molecules within the tissues as well as the protons in the amide groups within proteins are excited and saturated by a special saturation radiofrequency pulse (2.0 μT, 2 s). Through the chemical exchange between the amide protons and water protons, the free water in the tissue, which is equal to the amount of amide protons, is also saturated, leading to attenuation of the water signal. The degree of attenuation is positively correlated with the concentration of tissue amide protons. By detecting the change of the MRI signals before and after saturation, the concentration of amide protons in the tissue can be determined [10].

The diagnosis, treatment, and prognosis of BC are influenced by a variety of factors, such as lesion size, pathological grading, receptor expression, and so forth. These factors are related to the microscopic features of the lesions. Moreover, microscopic changes in metabolic status within BC tissues are much earlier than macroscopic changes in morphology. Since APTWI can provide microscopic information on the diseased tissue, such as the concentration and the change of amide protons [10], we surmised that APTWI can provide new ideas for the diagnosis, treatment, and prognostic assessment of early-stage BC.

Most studies on APTWI have focused on central nervous system diseases, where, for example, it has been used to distinguish high-grade from low-grade glioma [11], monitor the prognosis of glioma radiotherapy and chemotherapy [12], and distinguish postoperative recurrence and pseudoprogression [13]. At present, there are few studies on the application of APTWI to BC. APTWI studies of breast lesions have mostly focused on the differential diagnosis of benign and malignant lesions and the evaluation of treatment efficacy. To provide a brief overview, Klomp et al. [14] evaluated the reproducibility of APTWI at 7.0 T; Zaric et al. [15] explored the value of APTWI in the assessment of tumor grade and cell proliferation; Dula et al. [16] explored the role of APTWI in predicting the treatment efficacy of neoadjuvant chemotherapy; Krikken et al. [17] evaluated the reliability of APTWI in lymph node metastasis; and Wang et al. compared the value of APTWI *versus* intravoxel incoherent motion [18] or *versus* diffusional kurtosis imaging [19] in diagnosis and prognosis, respectively. The results of these studies have been inconsistent, and none has discussed the diagnostic value of APTWI in early-stage BC. Besides, most of the APTWI sequences used in previous studies were two-dimensional (2D) sequence, whereas in this study we used three-dimensional (3D) sequences. Indeed, the 3D sequence can obtain more layers of images in the same amount of time, which provides more accurate localization of the lesion and more information than that given by the 2D sequence.

The aim of this study was twofold: (1) to explore the diagnostic value of 3D-APTWI in early-stage BC by comparing the difference in magnetization transfer asymmetry (MTR_asym_) between benign and malignant breast lesions and (2) to analyze the correlations between parameters of 3D-APTWI and the immunohistochemical characteristics.

## Methods

### Study population

From November 2020 to August 2021, 117 female patients with breast lesions detected by mammography or/and ultrasonography were selected for standard breast MRI and 3D-APTWI; 69 patients underwent mammography and 60 patients underwent ultrasonography, 12 both mammography and ultrasonography. Clinical data and pathological results were collected.

The inclusion criteria were as follows: (1) successful MRI scan that yielded high-quality image meeting diagnostic requirements; (2) definitive pathological and immunohistochemical results were obtained by surgery or biopsy in the 2 weeks following MRI scanning.

**Fig. 1:**
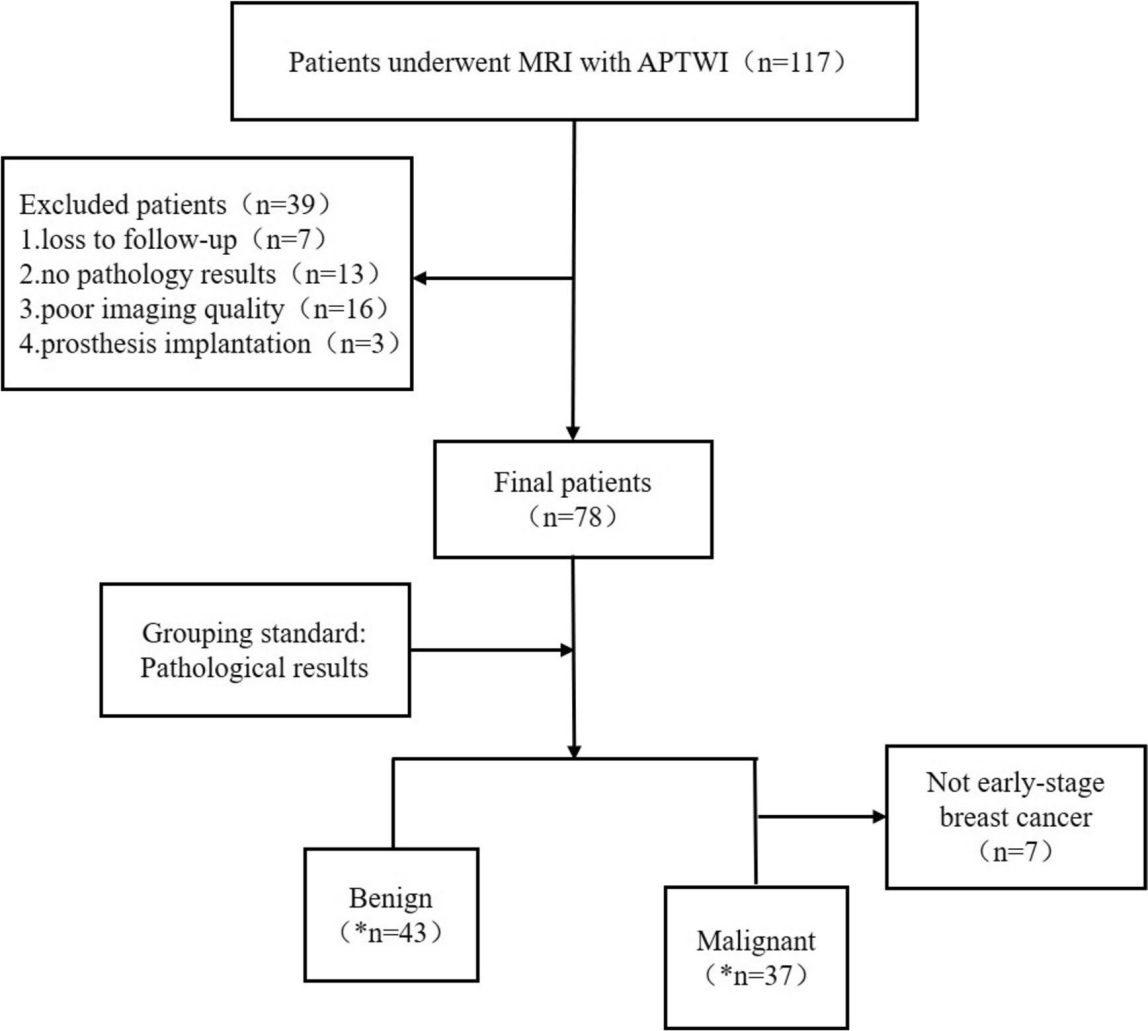
Flow chart of the patient selection process. *n* = number of patients *n = number of lesions

The exclusion criteria were as follows: (1) experienced prior therapy before MRI scan (needle biopsy, surgery, neoadjuvant chemotherapy, radiotherapy, chemotherapy, endocrine therapy, targeted therapies); (2) loss to follow-up; (3) post-prosthesis implantation (Fig. 1).

### Pathologic reference standard

All patients underwent needle biopsy or surgery to obtain lesion specimens, which were sent to the Department of Pathology of our hospital for analysis. Two pathologists independently analyzed the hematoxylin/eosin staining and immunohistochemistry results of the lesion specimens. The histopathological results were based on their consensus. Immunohistochemical results were used to determine the expression level of estrogen receptor (ER), progesterone receptor (PR), human epidermal growth factor receptor-2, and Ki-67.

### MRI protocol

Routine bilateral breast MRI was performed on a 3.0-T scanner (Ingenia CX, Philips Healthcare, Eindhoven, The Netherlands) with an 8-channel phased-array breast coil. Patients were placed in the prone position, with the foot entering the scanner first. Conventional imaging was performed, which included T1-weighted imaging, fat-suppressed T2-weighted imaging, and diffusion-weighted imaging. The APTWI sequences were planned such that enough slices would be taken to completely document the tumor tissue, using the images of the conventional sequence as a reference, under the guidance of an experienced radiologist. The pseudocolor images of the 3D-APTWIs are generated automatically after scanning. Patients were not to receive any contrast-enhanced examination within 24 h of the scanning to avoid interference with the APTWI signal [20]. Finally, a dynamic contrast-enhanced (DCE)-MRI scan was performed, 30 phases in total, with a 9.1-s scan time per phase [21, 22]. Gadopentetate dimeglumine was injected intravenously with a high-pressure syringe at a speed of 3.5 mL/s, a dose of 0.2 mL/kg, after the second phase. Following this, an equal amount of saline was injected at the same speed to rinse the pipe. In addition, a sagittal delayed contrast-enhanced scan was performed to assess the size and shape of axillary lymph nodes. All premenopausal patients were examined during the second week of the menstrual cycle, and there were no specific requirements for postmenopausal patients. The scanning sequence and parameters are shown in Table 1.

**Table 1:** Scanning parameters of the sequences

Parameters	T1WI	FS-T2WI	DWI	APTWI	DCE^*^
Orientation	Axial	Axial	Axial	Axial	Axial
Sequence name	FSE	FSE	SS-EPI	TSE-EPI	3D-VIBRANT
TR (ms)	569	4,057	12,500	5,864	3.8
TE (ms)	8	70	71	8.3	1.83
FOV (mm^2^)	280 × 380	280 × 380	340 × 255	230 × 180	200 × 310
Matrix	310 × 380	310 × 360	110 × 110	128 × 100	310 × 360
NSA	2	2	1	1	1
Slice thickness (mm)	4	4	4	4	4
Slice number	36	40	40	14	80^*^
Scan time (min:s)	2:13	2:58	2:30	6:32	4:59
Saturation pulse	—	—	—	2.0 μT	—
Saturation time (s)	—	—	—	2	—

^*^30 phases in total; 80 slices per phase. *APTWI* Amide proton transfer-weighted imaging, *DCE* Dynamic contrast enhanced, *DWI* Diffusion-weighted imaging, *FOV* Field of view, *FS-T2WI* Fat-suppressed T2 weighted imaging, *T1WI* T1-weighted imaging, *TR* Repetition time, *TE* Echo time, *NSA* Number of signal averages

### Data collection

All enrolled lesions were graded according to the American College of Radiology Breast Imaging Reporting and Data System−BI-RADS, 5^th^ edition. Category 4 and 5 lesions were classified as positive, and category 1 to 3 lesions were classified as negative. A breast radiologist with 15 years of experience, blinded to the pathological results, independently performed diagnostic categorization of all lesions. Lesions were divided into either the malignant or benign group based on their pathological results. Categorization of a sample as malignant was done according to the diagnostic standard for early BC [23]: (1) maximum lesion diameter of 2 cm; (2) absence of axillary lymph node metastasis; and (3) no distant metastasis.

Data postprocessing was performed by a radiologist with more than 3 years of experience under the supervision of a senior radiologist with 15 years of experience in breast MRI on a Philips workstation. Both were blinded to patients’ clinical history and other examination results. The pseudocolor images of the 3D-APTWIs were overlapped with the native contrast-enhanced images to identify the location of the lesions depicted therein. Subsequently, the region of interest (ROI) was manually delineated by outlining the contour of the tumor tissue using the native contrast-enhanced sequence as a reference. When drawing ROIs, necrosis, cystic changes, and bleeding areas were avoided as much as possible. Using these manually drawn ROIs, the workstation automatically calculated the MTR_asym_ value.

**Fig. 2:**
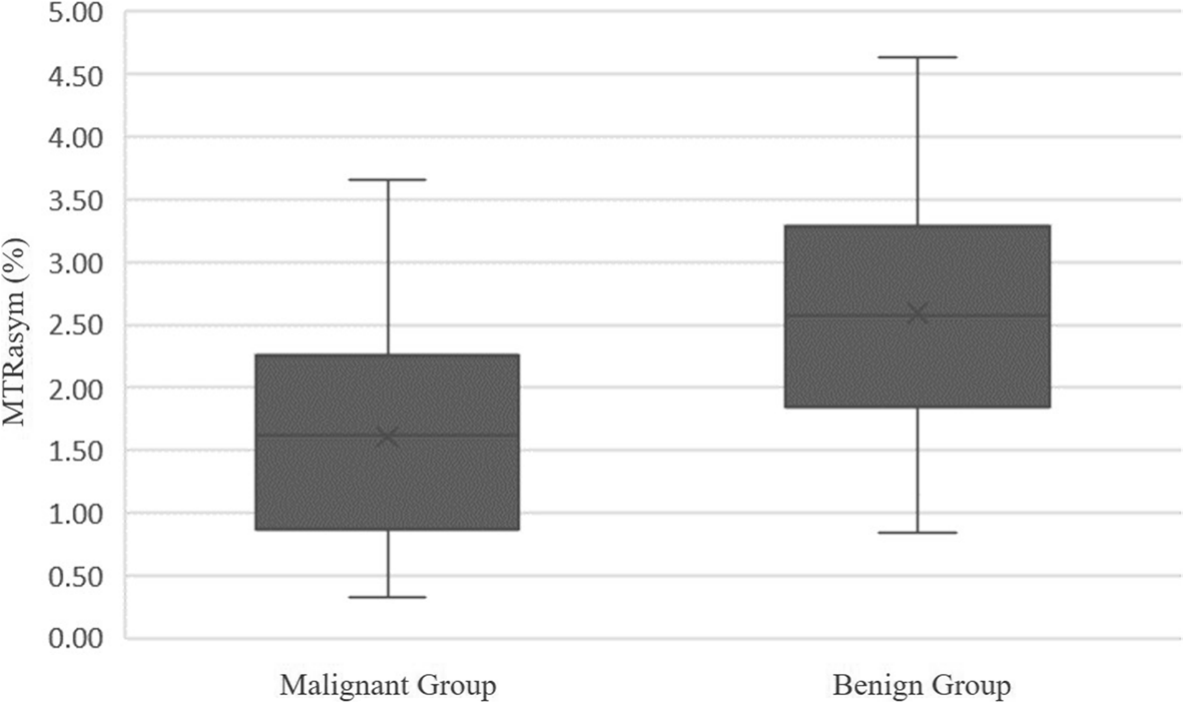
Box chart for magnetization transfer asymmetry (MTR_asym_) values in benign and malignant lesions

The DCE images were uploaded and analyzed by relevant software for pharmacokinetic analysis (Tissue 4D; Siemens Healthineers, Erlangen, Germany). Motion correction was performed automatically using the first dynamic image set as a reference. The ninth postcontrast dynamic dataset (acquired at 1 min after contrast injection) was used to draw the ROI. We draw the ROIs in the area, where was enhanced obviously. Pharmacokinetic evaluation was made based on the two-compartment Tofts model and a population average arterial input function (intermediate type) provided by Tissue 4D [24]. Within each ROI, we measured four quantitative properties: *K*_trans_ (volume transfer constant); *V*_*e*_ (volume fraction of extravascular space); *K*_ep_ (rate constant); and iAUC (initial area under the concentration-time curve). Time-intensity curves (TICs) were calculated for all ROIs. The semi-quantitative parameters included time-to-peak (TTP), PE (peak enhancement), wash-in rate, and wash-out rate.

### Statistical analysis

MedCalc 20.0 and SPSS 21.0 software were used for statistical analysis. The Kolmogorov-Smirnov test was used to evaluate whether the data of each group conformed to the normal distribution. Levene’s test was used to assess the equality of variances. Independent sample *t*-test and χ^2^ test were used to analyze and compare the difference in the values of parameters between the benign and malignant groups. A binomial logistic regression model was established for each parameter, and statistically significant parameters were selected to establish a multi-parameter diagnostic model. The predicted probability of logistic regression was used to generate a receiver operating characteristic (ROC) curve to evaluate diagnostic performance of APTWI, DCE, and APTWI+DCE by each AUC. DeLong’s test was used to analyze whether there are differences in the areas under the ROC curve (ROC-AUCs) of each parameter. Pearson’s correlation coefficient was used to analyze the correlation between MTR_asym_ value and Ki-67. *p*-values < 0.05 were considered statistically significant.

## Results

### Clinical features

The patient screening process is shown in Fig. 1. Our study cohort consisted of 78 female patients, aged 14–18 years (52 ± 7.1, mean ± standard deviation). Clinical features of the enrolled patients and TIC of the lesions are shown in Table 2. Patients in the benign group were significantly younger than those in the malignant group. Among these patients, four had multiple lesions in one breast. The largest of these were selected for this study, which provided various indicators and were included in the group corresponding to pathological results. Another four patients had one benign in one breast and one malignant lesion in the other breast; in these cases, the benign and malignant lesions were included separately [25]. Moreover, three patients had benign lesions in both breasts, and two patients had malignant lesions in both breasts; in these cases, each lesion was evaluated separately. All patients underwent mammography or/and ultrasonography before MRI. The details about lesions are shown in Table 2.

**Table 2:** Characteristics of patients/lesions and time-signal intensity curves

	Benign lesions (*n* = 43)	Malignant lesions (*n* = 37)	*p*-value	Sensitivity (%)	Specificity (%)
Age (years)	45.0 ± 2.8	52.5 ± 6.4	< 0.010	—	—
Size (cm) (range)	1.09 ± 2.09 (0.3−9.4)	1.29 ± 0.35 (0.2−2.0)	—	—	—
TIC type [*n* (%)]			< 0.010	—	—
Wash-in/type I	26 (60.5%)	6 (16.2%)	—	—	—
Plateau/type II	9 (20.9%)	10 (27.0%)	—	83.8	60.5
Wash-out/type III	8 (18.6%)	21 (56.8%)	—	56.7	81.4
The part below must be modified					
Mammography					
Mass	13 (44.8%)	16 (55.2%)	—	—	—
Focal asymmetry	9 (60.0%)	6 (40.0%)	—	—	—
Amorphous calcifications	2 (12.5%)	14 (87.5%)	—	—	—
Architectural distortion	5 (38.5%)	8 (61.5%)	—	—	—
Fibrocystic changes	12 (100.0%)	0 (0.0%)	—	—	—
Ultrasonography					
Mass	20 (46.5%)	23 (53.5%)	—	—	—
Irregular hypoechoes	8 (47.1%)	9 (52.9%)	—	—	—
Strong echoes	6 (26.1%)	17 (73.9%)	—	—	—
Mammary duct ectasia	3 (60.0%)	2 (40.0%)	—	—	—

*TIC* Time signal-intensity curve

A total of 80 lesions were included in this study, of which 43 were in the benign group. These included 20 fibrocystic changes, 12 fibroadenomas, 5 intraductal papillomata, 2 benign phyllode tumors, 1 foreign body granuloma, and 1 hamartoma. Of 44 malignant lesions, 7 were non-early BC and were excluded. The remaining 37 early BCs consisted of 17 invasive ductal carcinomas, 9 ductal carcinomas *in situ*, 6 mixed carcinomas, 2 encapsulated papillary carcinomas, 2 invasive lobular carcinomas, and 1 mucinous carcinoma.

### Parameter comparison

A comparison of the parameters of the benign group *versus* the malignant group is shown in Table 3 and Fig. 2. The MTR_asym_, V_e_, and TTP of the malignant group were significantly lower than those of the benign group with statistical (1.19 ± 0.82% [mean ± standard deviation] *versus* 2.68 ± 1.19%, *p* < 0.010; 0.26 ± 0.10 *versus* 0.34 ± 0.22, *p* = 0.321; 162.54 ± 75.00 s *versus* 227.08 ± 72.29 s, *p* < 0.010). The *K*_trans_, *K*_ep_, wash-in rate, and wash-out rate of the malignant group were all significantly greater than those of the benign group (0.26 ± 0.19 min^-1^ *versus* 0.13 ±0.08 min^-1^, *p* < 0.010; 1.09 ± 0.87 min^-1^ *versus* 0.48 ± 0.33 min^-1^, *p* < 0.010; 102.22 ± 51.66% *versus* 67.87 ± 43.98%, *p* < 0.010; 15.70 ± 14.28% *versus* 9.52 ± 10.96%, *p* = 0.034. The differences in the other quantitative parameters among the benign and malignant groups were not statistically significant (all *p* > 0.050).

**Table 3:** Comparison of different parameters between benign and malignant lesions

	Benign lesions (*n* = 43)	Malignant lesions (*n* = 37)	*p*-value	ROC-AUC (95% CI)
Parameters from APTWI				
MTR_asym_ (%)	2.7 ± 1.2	1.2 ± 0.8	< 0.01	0.816 (0.724–0.909)
Semiquantitative parameters				
PE (%)	158.9 ± 71.0	186.6 ± 63.6	0.06	0.628 (0.508–0.748)
TTP (s)	227.1 ± 72.3	162.5 ± 75.0	< 0.01	0.727 (0.616–0.838)
Wash-in rate (%)	67.9 ± 44.0	102.2 ± 51.7	< 0.01	0.699 (0.587–0.810)
Wash-out rate (%)	9.5 ± 11.0	15.7 ± 14.3	0.03	0.665 (0.547–0.782)
Pharmacokinetic parameters				
K_trans_ (min^−1^)	0.1 ± 0.1	0.3 ± 0.2	< 0.01	0.737 (0.619–0.834)
*K*_ep_ (min^−1^)	0.5 ± 0.3	1.1 ± 0.9	< 0.01	0.746 (0.628–0.842)
*V*_*e*_	0.3 ± 0.2	0.3 ± 0.1	0.32	0.580 (0.457–0.696)
iAUC (×10^−3^ mm^2^/s)	0.1 ± 0.1	0.2 ± 0.1	0.06	0.633 (0.510–0.744)

*APTWI* Amide proton transfer-weighted imaging, *ROC-AUC* Area under the receiver operating characteristic curve, *iAUC* Initial area under the gadolinium concentration-time curve, *K*_*ep*_ Rate constant, *K*_*trans*_ Volume transfer constant, *MTR*_*asym*_ Magnetization transfer asymmetry, *PE* Peak enhancement, *TTP* Time to peak, *V*_*e*_ Extravascular extracellular volume fraction

The immunohistochemical features of BC are shown in Table 4. The MTR_asym_ value was significantly higher in the high-grade pathology group than that in the low-grade pathology group (2.18 ± 0.76% *versus* 1.30 ± 0.69%, *p* = 0.004). The MTR_asym_ value of the ER-positive group and the ER-negative group were almost identical (1.61 ± 0.83% *versus* 1.60 ± 0.85%, *p* = 0.98). Differences in MTR_asym_ value between the other groups did exist, but lacked statistical significance (*p* ≥ 0.050).

**Table 4:** Immunohistochemical characteristics of breast cancers

	Number of lesions [*n* (%)]	MTR_asym_ (%)	*p-*value
Histologic grades			0.004
High	25 (67.57)	2.18 ± 0.76	
Low	12 (32.43)	1.30 ± 0.69	
ER			0.964
Positive	32 (86.49)	1.61 ± 0.83	
Negative	5 (13.51)	1.60 ± 0.85	
PR			0.323
Positive	30 (81.08)	1.67 ± 0.81	
Negative	7 (18.92)	1.32 ± 0.86	
Her-2			0.230
Positive	14 (37.84)	1.40 ± 0.90	
Negative	23 (61.16)	1.73 ± 0.82	
Ki-67			0.286
High-expression	24 (64.86)	1.73 ± 0.85	
Low-expression	13 (35.14)	1.37 ± 0.74	

*ER* Estrogen receptor, *HER-2* Human epidermal growth factor receptor-2, MTR_asym_ Magnetization transfer asymmetry, *PR* Progesterone receptor

### Diagnostic efficiency and correlation analysis

The difference in the distribution of the time-signal intensity curve (TIC) between early BC and benign lesions was statistically significant (*χ*^2^ = 18.032, *p* < 0.001). When the type II and III curves were used as the diagnostic criteria for malignant lesions, the sensitivity was 83.8%, and the specificity was 60.5%. When the type III curve was considered as the criteria, the sensitivity was 56.8% and the specificity was 81.4% (Table 2).

**Fig. 3:**
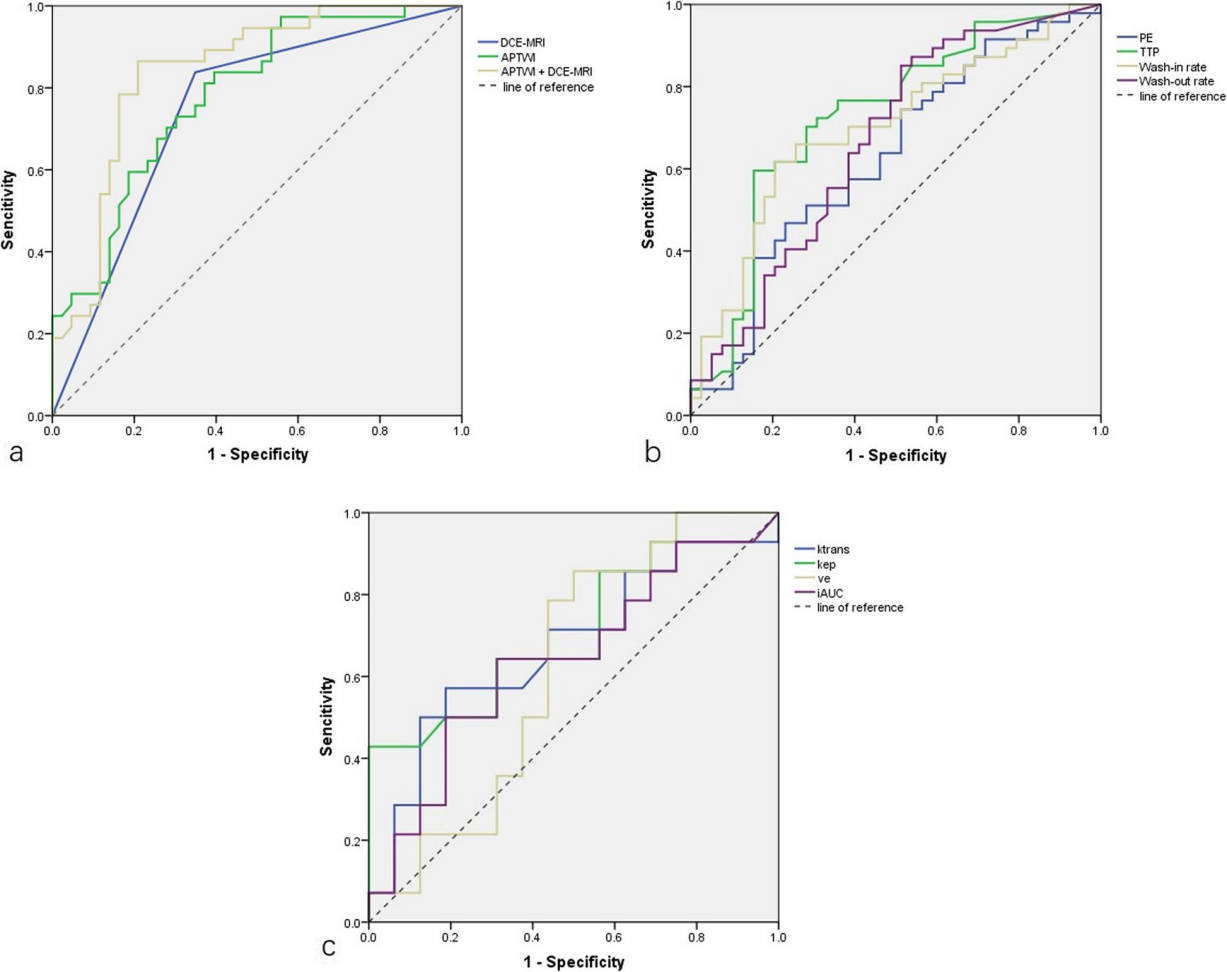
Receiver operating characteristic curves of parameters and methods for discriminating early breast cancer and benign lesions

**Fig. 4:**
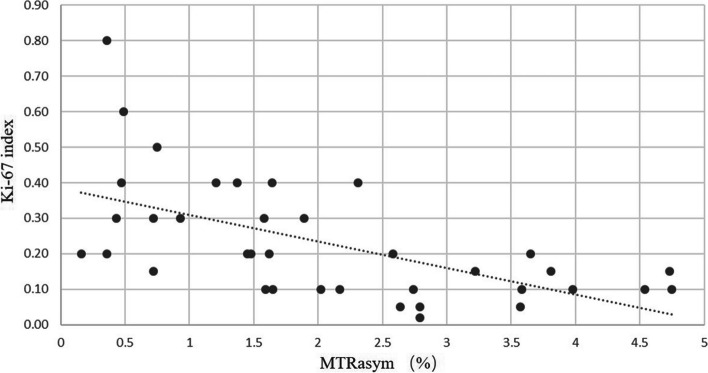
The correlation between magnetization transfer asymmetry (MTR_asym_) and Ki-67

Figure 3 shows the ROC curves of parameters and methods for discriminating early BC from benign lesions. When the diagnostic threshold of MTR_asym_ was 2.42%, the sensitivity and specificity were 86.5% and 67.6%, respectively. Considering the diagnosis of malignant *versus* benign lesions, the ROC-AUC_*K*trans_>ROC-AUC_*K*ep_>ROC-AUC_iAUC_>ROC-AUC_Ve_, ROC-AUC_TTP_> ROC-AUC_wash-in_>ROC-AUC_wash-out_>ROC-AUC_PE_, but the differences among them were not significant (all *p* > 0.050). According to different methods, the difference between ROC-AUC_APTWI+DCE_ and ROC-AUC_DCE_ (0.858 *versus* 0.745, 95% confidence interval 0.770–0.945 *versus* 0.634–0.855) was significant (*Z* = 2.785, *p* < 0.010). Hence, 3D-APTWI showed equal efficiency in diagnosing early BCs from benign lesions as DCE-MRI and can add diagnostic accuracy to it when combined together. In addition, ROC-AUC_3D-APTWI_ (0.816, 95% confidence interval 0.724−0.909) was higher than ROC-AUC_DCE_, but without statistical significance (*Z* = 1.617, *p* = 0.106). According to Pearson’s correlation analysis, the MTR_asym_ value of the malignant group showed a modestly positive correlation with the Ki-67 (22.9 ± 16.9%, *r* = 0.419, *p* = 0.020) (Fig. 4).

## Discussion

In this study, we mainly compared the parameters of 3D-APTWI and DCE-MRI between early BC and benign lesions and analyzed the correlation between these parameters and the immunohistochemical characters. Our data highlight three main findings:

1. We found that the MTR_asym_ value of early BC was lower than that of the benign lesions:
2. We were able to use 3D-APTWI to differentially diagnose early BC from benign lesions, potentially increasing the diagnostic efficacy of DCE-MRI;
3. The MTR_asym_ value was associated with the proliferation of BC.

It is well known that breast tissue has a strong secretory function under normal conditions. The metabolism of malignant tumor tissue cannot compensate for the destruction of normal secretory function, and the content of proteins and peptides is thus reduced. Thus, the MTR_asym_ value of malignant lesions is lower than that of benign lesions, which was consistent with previous studies [11, 15, 19]. However, the object of our study, early-stage BC, has not been the focus of previous studies.

**Fig. 5:**
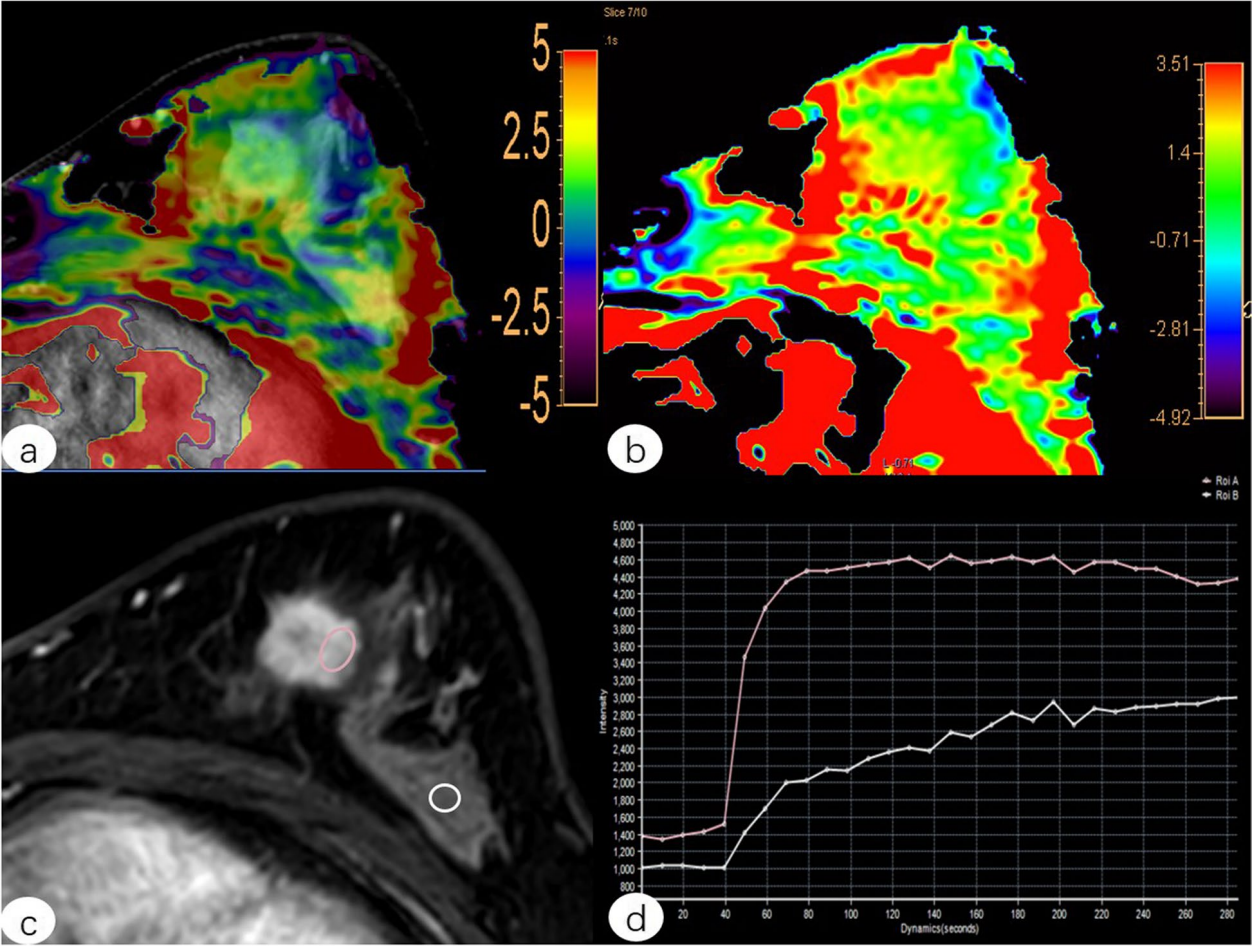
Female, 48-year-old, with invasive ductal carcinoma in the left breast. **a** Fused image of **b** and **c**. **b** Pseudo-color picture of APTWI (MTR_asym_ = 1.45%). **c** Postcontrast axial image. **d** Time-signal intensity curve type III. *ROI A (red line): lesion, ROI B (white line): ipsilateral normal breast tissue. *APTWI* Amide proton transfer-weighted imaging, *MTR*_*asym*_ Magnetization transfer asymmetry, *ROI* Region of interest

**Fig. 6:**
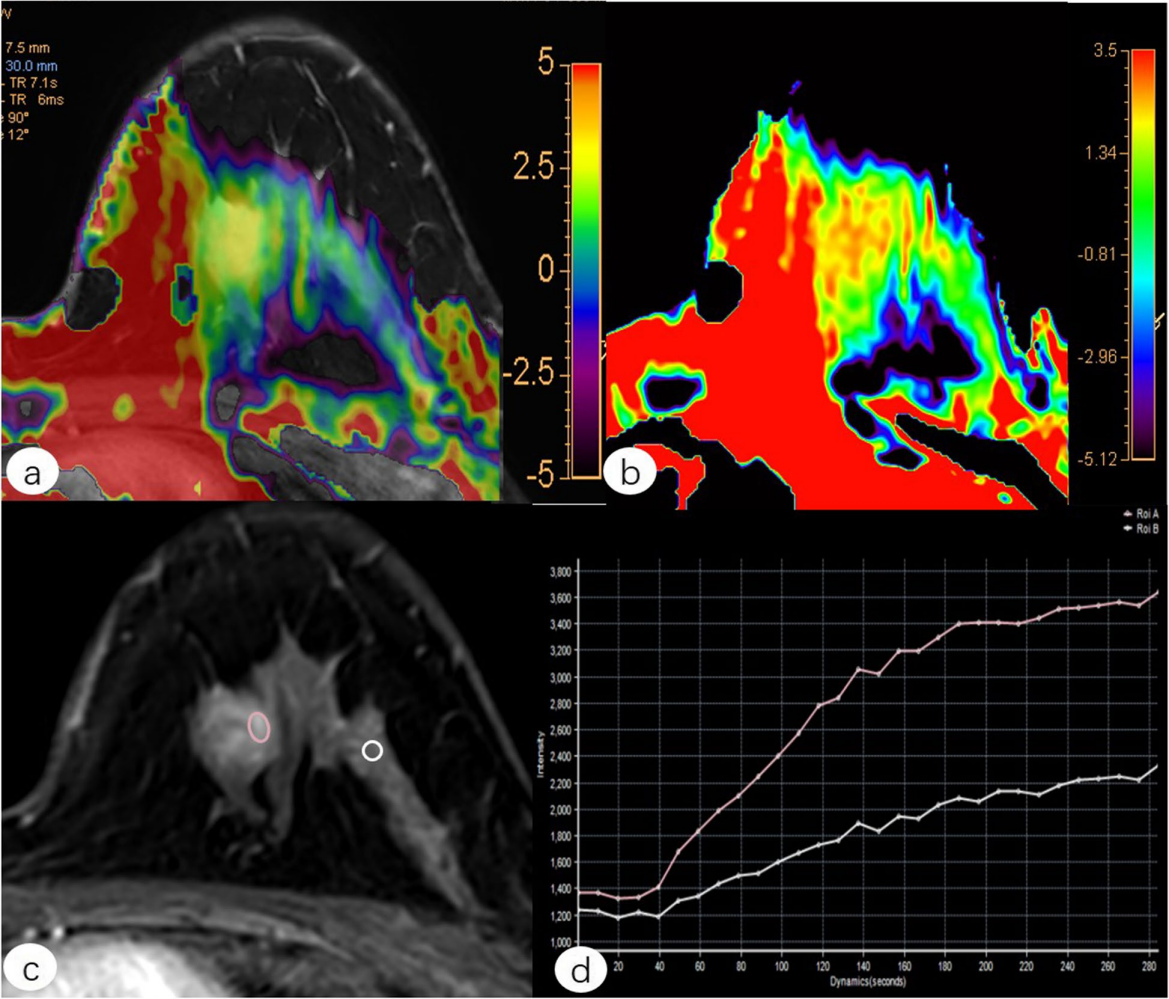
Female, 36-year-old, with a fibroadenoma in the left breast. **a** Fused image of **b** and **c**. **b** Pseudo-color picture of APTWI (MTR_asym_ = 3.57%). **c** Postcontrast axial image. **d** Time-signal intensity curve type I. *ROI A (red line): lesion, ROI B (white line): ipsilateral normal breast tissue. *APTWI* Amide proton transfer-weighted imaging, *MTR*_*asym*_ Magnetization transfer asymmetry, *ROI* Region of interest

At present, it is still a challenging task for clinicians to diagnose BC at an early stage. Our study demonstrates the potential of APTWI in the diagnosis of early-stage BC. We were able to distinguish early BC from benign lesions by 3D-APTWI, suggesting that 3D-APTWI might be used as a complementary method when a DCE-MRI scan cannot be performed due to severe renal insufficiency or other reasons. We subsequently found that 3D-APTWI combined with DCE-MRI provided the largest ROC-AUC for discriminating early BC and benign lesions, with the difference between AUC_DCE-MRI_ and AUC_APT+DCE_ being statistically significant. Thus, the combination of 3D-APTWI and DCE-MRI can effectively improve the diagnostic accuracy of breast lesions (Figs. 5 and 6).

In this study, the *K*_trans_ and *K*_ep_ values of malignant lesions were higher than those of the benign lesions, and the V_e_ value of malignant lesions was lower than that of the benign lesions, both with statistical significance and consistent with previous studies [26, 27], supporting the diagnostic value of DCE-MRI. Li et al. [28] showed that the AUC of both *K*_trans_ and *K*_ep_ in diagnosing benign and malignant breast lesions ranged from 0.7 to 0.9, whereas in Cheng et al. [29], the AUC of both *K*_trans_ and *K*_ep_ in diagnosing benign and malignant breast lesions was above 0.9. In the present study, the AUCs of *K*_trans_ and *K*_ep_ were 0.737 and 0.746, respectively, which were slightly lower than those reported by Cheng et al. [29]. We speculate that this may be related to the lower vascular permeability of early BC compared with that of advanced BC. Singh et al. [30] observed in artificially prepared 3D tumor microenvironments that advanced BC microenvironments exhibit hypoxia, upregulation of vascular endothelial growth factor expression, enhanced angiogenesis, and basal membrane degradation, and thus higher vascular permeability in advanced BC. However, whether both the differences in microcirculation and vascular permeability between early and advanced BCs are similar in humans requires large clinical samples and basic experiments to further confirm.

iAUC refers to the amount of change in signal intensity over time when the contrast agent reaches and stays in the tissue and blood vessels, which can reflect the blood volume of the target tissue. The difference in iAUC for the differential diagnosis of benign and malignant breast lesions was statistically significant in the study of Cheng et al. [29]. However, iAUC showed no diagnostic significance in distinguishing benign breast lesions from early BC in our study. This result was different from that reported by Cheng et al. [29]. Petit et al. [31] found a reduction in vascular endothelial production factor after treatment of BC cells with human epidermal growth factor receptor-2 (Her-2) antibody, suggesting that Her-2 is associated with the expression and secretion of angiogenic factors. Her-2 can stimulate tumor angiogenesis by up-regulating angiogenic growth factors, and this effect is further enhanced under hypoxic conditions. In the present study, the proportion of patients with positive Her-2 expression was very low, only about one-third (14/37). This may be due to the low level of Her-2 positivity in this study, resulting in a smaller difference in blood volume between early BC and benign breast lesions. Considering the small sample size of this study, it is still necessary to conduct an in-depth study of this issue in the future.

This study shows that the MTR_asym_ value was slightly correlated with the Ki-67 expression. The higher the expression level of Ki-67, the more active the proliferation of tumor cells and the worse the prognosis of the patient [32]. Ki-67 is an important indicator when formulating chemotherapy regimens for breast cancer [33], which is of great significance in improving prognosis and quality of life. However, this result was not completely consistent with previous studies. For example, Zaric et al. [15] concluded that MTR_asym_ values were highly positively correlated with the Ki-67, while Wang Meiyun et al. [18] suggested that MTR_asym_ value showed a low positive correlation with Ki-67. Given the difference in sample content, field strengths, and scanning machines, we speculate that MTR_asym_ values could be used for preliminary assessment of the Ki-67 expression level in BC patients, APTWI has the potential to assess the cell proliferation of BC. However, the relationship between the MTRasym value and the Ki-67 proliferation index needs to be studied in depth.

The 3D-APTWI sequence used in this study is a product sequence launched by Philips Heathcare, which can perform multilayer scanning in a relatively short scanning time (about 6 min 32s) and provide more images. However, most of the APTWI sequences used in the published research on breast lesions are 2D sequences [18, 19]. Single-layer acquisition may not cover the whole lesion and obtain the APT value accurately, even in early-stage BC with small lesions. The 3D sequence can generate pseudo-color images after scanning directly without manually postprocessing, which is kind for radiologists and time-saving. The MTR_asym_ value can be obtained directly by drawing ROIs on the image, which is simpler and more convenient than using a 2D sequence. Compared to sequences used in the previous studies that required multi-step post-processing, the clinical applicability of the commercial sequence used in the current study should be more widely accepted.

The main limitations of this study are as follows:

First, a relatively small number of patients was included in this study, especially when the different immunopathology was taken into account; as such, the results may not be broadly representative. Larger sample sizes will be needed to properly correlate APTWI with various immunohistochemical parameters in the future. Second, the APTWI sequences have relatively poor spatial resolution, making it difficult to clearly resolve tiny lesions less than 5 mm in diameter, affecting the accuracy of this study to some extent. This is limited by contemporary machines and needs further study. Third, the breast tissue contains more or less abundant fat, and this leads to some lesions located at the edges of the glands and inside fat deposits showing poor resolution. In addition, the adipose tissue and normal gland tissue did not show exact images or negative values on the APT pseudocolor image in some patients with breast hyperplasia. It was discovered that the breast tissues in these patients were all less glandular or fatty. The fibroglandular and adipose tissues of the breast are intertwined. Therefore, we speculate that this may be due to the presence of a large number of lipid signals that affect the chemical exchange saturation transfer−CEST effect and produce artifacts [34], which need further study. Finally, this study did not combine the DCE sequence with other sequences, such as T2WI and DWI, and did not use other methods integrating DCE with unenhanced sequences, which we will do extensive study in the future.

In conclusion, in our study 3D-APTWI showed potentials to be helpful in differential diagnosis of benign breast lesions and early-stage BC. 3D-APTWI combined with DCE-MRI can improve the diagnostic accuracy of early-stage BC.

## Data Availability

The datasets used and/or analyzed during the current study are available from the corresponding author on reasonable request. We declare that we have not used a Large Language Models (LLMs) for our manuscript.

## Abbreviations

2D: Two-dimensional
3D: Three-dimensional
APTWI: Amide proton transfer-weighted imaging
BC: Breast cancer
DCE: Dynamic contrast-enhanced
ER: Estrogen receptor
HER-2: Human epidermal growth factor receptor-2
iAUC: Initial area under the gadolinium concentration-time curve
*K*_ep_: Rate constant
*K*_trans_: Volume transfer constant
MTR_asym_: Magnetization transfer asymmetry
PE: Peak enhancement
PR: Progesterone receptor
ROC: Receiver operating characteristic
ROC-AUC: Areas under the ROC curve
ROI: Region of interest
TIC: Time-intensity curve
TTP: Time-to-peak
*V*_*e*_: Volume fraction of extravascular space
